# Concomitant abdominal organ transplantation alongside lung transplantation: An ISHLT transplant database analysis

**DOI:** 10.1016/j.jhlto.2024.100200

**Published:** 2024-12-20

**Authors:** Renita Wilson, J. Asher Jenkins, Juan Maria Farina, Blake Langlais, Bashar Aqel, Ashraf Omar, Jonathan D’Cunha, Pedro Reck dos Santos

**Affiliations:** a Mayo Clinic Alix School of Medicine, Phoenix, Arizona; b Department of Cardiothoracic Surgery, Mayo Clinic Arizona, Phoenix, Arizona; c Division of Clinical Trials and Biostatistics, Mayo Clinic Arizona, Phoenix, Arizona; d Division of Gastroenterology and Hepatology, Mayo Clinic Arizona, Phoenix, Arizona; e Division of Transplant Pulmonology, Mayo Clinic Arizona, Phoenix, Arizona

**Keywords:** concomitant transplant, lung transplant, abdominal transplant, graft survival, simultaneous transplant

## Abstract

**Background:**

Concomitant abdominal organ transplant of the liver, kidney, and/or pancreas with lung transplant (Con-AbLTx) may be considered for appropriate patients who present with end-stage disease of multiple organ systems. Most existing literature examines outcomes of combined lung-liver transplants, with little attention paid to other commonly transplanted abdominal organs, such as kidneys and pancreas. This study aims to examine post-transplant outcomes of patients submitted to Con-AbLTx to lung transplant (LTx)-only recipients.

**Methods:**

The international society for heart and lung transplantation (ISHLT) International Thoracic Organ Transplant Registry for Con-AbLTx and LTx-only was reviewed from January 1994 to June 2018. LTx-only recipients were propensity score matched 4:1 based on various patient characteristics. Data were analyzed with Fisher’s exact, Wilcoxon rank sum tests, Kaplan-Meier methods, and Cox proportional hazards where appropriate.

**Results:**

A total of 195 Con-AbLTx and 780 propensity-matched LTx-only cases were compared. LTx-only recipients demonstrated higher levels of bronchiolitis obliterans syndrome. Following transplant, Con-AbLTx required a longer hospital stay and post-transplant dialysis before discharge. LTx-only were more likely to experience graft failure from acute rejection or chronic rejection. Con-AbLTx experienced higher 1-year mortality than LTx-only counterparts, with the highest mortality seen in the concomitant lung/kidney group. Of concomitant transplants, lung/liver recipients had greater survival over time.

**Conclusions:**

Con-AbLTx has the potential to carry substantial morbidity. At 10 years post-transplant, there is no statistically significant difference in survival between LTx-only and Con-AbLTx recipients. Given limited organ availability and ethical considerations of simultaneous transplant, careful consideration for Con-AbLTx is paramount to achieve acceptable outcomes.

## Background

Lung transplantation (LTx) is the definitive treatment for patients with end-stage lung disease (ESLD). Patients in whom their end-stage lung disease is severe enough to require LTx are often complex, with multiple organ systems being affected due to the interplay between pulmonary, cardiac, and abdominal solid organ physiology both before and after transplantation. For example, diseases such as cystic fibrosis (CF) and alpha-1 antitrypsin deficiency (A1AT) affect abdominal organs in addition to the lungs and may require consideration for subsequent abdominal organ transplant to definitively treat their primary disease process.^1^, ^2^, ^3^, ^4^ Other patients may have separate etiologies for lung and abdominal organ dysfunction which may also necessitate simultaneous transplantation of more than one organ.

Post-transplant, use of immunosuppressive medications can result in severe renal dysfunction, a leading cause of morbidity and mortality after lung transplant.^5^ Such patients may become potential candidates for subsequent kidney transplant. Thus, for select patients with systemic diseases affecting multiple organ systems or complex multiorgan illnesses, transplantation of a concomitant abdominal organ alongside LTx (Con-AbLTx) may be beneficial in addressing multiorgan dysfunction.

Much of the existing literature pertains to simultaneous liver/lung transplant and is widely variable in terms of survival outcomes. Concomitant liver/lung transplant has demonstrated inferior survival when compared to liver transplant alone.^6^ When compared to lung transplant alone, combined liver/lung recipients have been observed to experience similar survival outcomes at 5 years post-transplant.^6^, ^7^ Other studies evaluating simultaneous lung/kidney transplants have reported improved long-term survival in dual-organ recipients compared to lung-only recipients.^8^ However, a broader analysis of thoracic/abdominal organ transplant compared to thoracic-only transplants showed no significant survival difference between the 2 groups.^9^

Although there are several single-center analyses on simultaneous lung/liver transplants and database reports regarding outcomes for thoracic/abdominal organ transplant, these results are widely variable in terms of survival outcomes. This study aims to provide a comprehensive analysis of the characteristics and outcomes of patients who undergo Con-AbLTx.

## Materials and methods

### IRB statement

This study was deemed exempt from formal review by the Mayo Clinic Institutional Review Board as all data was deidentified before receipt. The authors agree with and confirm that their study adheres to the principles of the World Medical Association Statement on Organ and Tissue Donation, the Declaration of Helsinki, and the Declaration of Istanbul.

### Study data

**Figure 1:**
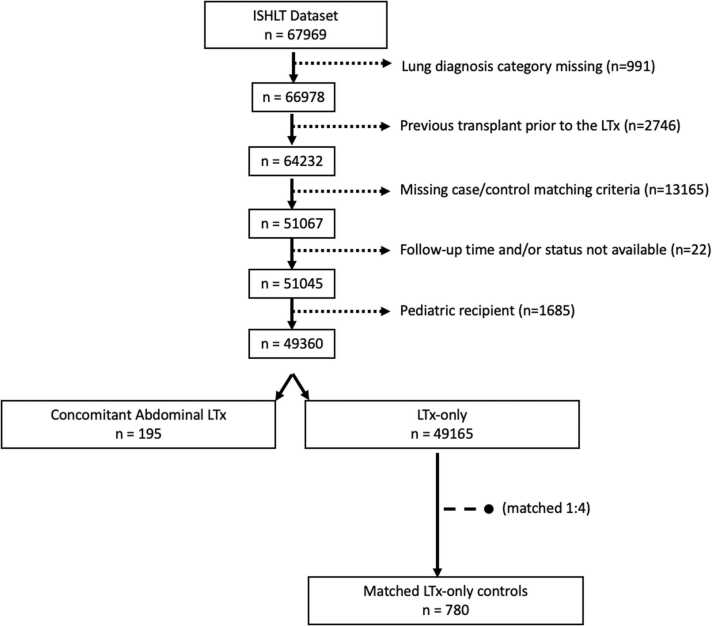
Consort diagram. LTx, lung transplantation.

The international society for heart and lung transplantation (ISHLT) Thoracic Organ Transplant database as of April 19, 2019 was queried for patients who underwent LTx, as well as patients who underwent Con-AbLTx from January 1, 1994 to June 30, 2018. In the context of this dataset, a “simultaneous transplant” refers to transplants of multiple organs from the same donor to the same recipient with the exclusion of kidney-pancreas and heart-lung transplants. Propensity score matching was used to create a comparator control group to the Con-AbLTx group. Possible controls included LTx-only recipients meeting the study criteria (Figure 1). LTx-only controls were propensity score matched 1:4 (case:control) using a standard set of characteristics, including age, sex, body mass index, end-stage lung disease category, and year of LTx. Propensity scores were constructed using logistic regression and nearest neighbors were matched without replacement. Matching was carried out using the R language and environment for statistical computing (v4.3.2) and the matchit package (v4.5.5).^10^, ^11^ Severe renal dysfunction was defined as creatinine >2.5 mg/dl, dialysis requirement, or renal transplant.

### Statistical analysis

Fisher's exact tests were used to compare frequency distributions between groups. Nonparametric Wilcoxon rank sum tests compared continuous measures alongside medians and interquartile ranges (IQR). Kaplan-Meier methods were used for time-to-event estimates and corresponding confidence intervals (including overall and graft-failure–free survival). Log-rank tests compared survival distributions. Unadjusted Cox proportional hazards models were considered to estimate hazard ratios between groups where model assumptions permitted their use. *p*-Values less than 0.05 were deemed statistically significant.

## Results

A total of 195 Con-AbLTx matched cases and 780 propensity-matched controls were identified. The Con-AbLTx cases consisted of 135 (69.2%) lung/liver, 51 (26.2%) lung/kidney, 4 (2.1%) lung/kidney/liver, 3 (1.5%) lung/pancreas, and 2 (1.0%) lung/liver/pancreas. The frequency of cases over time is demonstrated in Table 1.

**Table 1:** Frequency of Transplant Cases by Year

Transplant year	Con-AbLTx (*n* = 195)	LTx-only (*n* = 780)	Total (*n* = 975)
1994	2 (1.0%)	11 (1.4%)	13 (1.3%)
1995	3 (1.5%)	14 (1.8%)	17 (1.7%)
1996	2 (1.0%)	12 (1.5%)	14 (1.4%)
1997	3 (1.5%)	13 (1.7%)	16 (1.6%)
1998	4 (2.1%)	13 (1.7%)	17 (1.7%)
1999	5 (2.6%)	22 (2.8%)	27 (2.8%)
2000	5 (2.6%)	18 (2.3%)	23 (2.4%)
2001	3 (1.5%)	12 (1.5%)	15 (1.5%)
2002	2 (1.0%)	11 (1.4%)	13 (1.3%)
2003	7 (3.6%)	31 (4.0%)	38 (3.9%)
2004	4 (2.1%)	15 (1.9%)	19 (1.9%)
2005	3 (1.5%)	19 (2.4%)	22 (2.3%)
2006	12 (6.2%)	53 (6.8%)	65 (6.7%)
2007	11 (5.6%)	39 (5.0%)	50 (5.1%)
2008	4 (2.1%)	13 (1.7%)	17 (1.7%)
2009	16 (8.2%)	52 (6.7%)	68 (7.0%)
2010	8 (4.1%)	37 (4.7%)	45 (4.6%)
2011	13 (6.7%)	57 (7.3%)	70 (7.2%)
2012	7 (3.6%)	26 (3.3%)	33 (3.4%)
2013	10 (5.1%)	44 (5.6%)	54 (5.5%)
2014	7 (3.6%)	32 (4.1%)	39 (4.0%)
2015	20 (10.3%)	80 (10.3%)	100 (10.3%)
2016	14 (7.2%)	55 (7.1%)	69 (7.1%)
2017	21 (10.8%)	67 (8.6%)	88 (9.0%)
2018	9 (4.6%)	34 (4.4%)	43 (4.4%)

Abbreviations: Con-AbLTx, concomitant abdominal organ transplant of the liver, kidney, or pancreas with lung transplant; LTx, lung transplantation.

Patient characteristics are outlined in Table 2. The median age of Con-AbLTx transplants was 37 (IQR 26-52) years and the median age of LTx-only recipients was 33 (IQR 25-51) years. In addition, 65 (33.3%) of Con-AbLTx recipients were female with 252 (32.3%) females in the LTx-only group. The most common lung diagnosis among both groups was CF, accounting for 96 (49.2%) of Con-AbLTx and 409 (52.4%) LTx-only cases (Table 3).

**Table 2:** Characteristics of Con-AbLTx Recipients and LTx Controls

Variable	Con-AbLTx (*n* = 195)	LTx-only (*n* = 780)	Total (*n* = 975)	*p*-value
Concomitant organ, *n* (%)				-
LTx-only	-	780 (100.0%)	780 (80.0%)	
Lung+kidney	51 (26.2%)	-	51 (5.2%)	
Lung+kidney+liver	4 (2.1%)	-	4 (0.4%)	
Lung+liver	135 (69.2%)	-	135 (13.8%)	
Lung+liver+pancreas	2 (1.0%)	-	2 (0.2%)	
Lung+pancreas	3 (1.5%)	-	3 (0.3%)	
Recipient				
Age, median (IQR)	37 (26-52)	33 (25-51)	34 (25-51)	-
BMI (kg/m^2^), median (IQR)	20.4 (18.2-24.9)	20.2 (18.0-24.3)	20.2 (18.0-24.3)	-
Female, *n* (%)	65 (33.3%)	252 (32.3%)	317 (32.5%)	-
Lung diagnosis category, *n* (%)				
A1ATD	10 (5.1%)	36 (4.6%)	46 (4.7%)	
CF	96 (49.2%)	409 (52.4%)	505 (51.8%)	
COPD	9 (4.6%)	39 (5.0%)	48 (4.9%)	
IIP	34 (17.4%)	133 (17.1%)	167 (17.1%)	
IPAH	13 (6.7%)	48 (6.2%)	61 (6.3%)	
Pretransplant				
Chronic steroid use, *n* (%)^a^	36 (30.3%)	180 (37.7%)	216 (36.2%)	0.1372^b^
Cigarette pack-years, median (IQR)^c^	5 (2-6)	2.5 (2-4)	3 (2-5)	0.1293^d^
History of diabetes, *n* (%)^e^	48 (38.2%)	150 (28.3%)	198 (30.3%)	0.1384^b^
Recent hospitalization within 90 days of transplant admission, *n* (%)^f^	23 (43.4%)	100 (47.4%)	123 (46.6%)	0.6460^b^
Recent serum creatinine (mg/dl), median (IQR)^g^	0.9 (0.6-1.4)	0.8 (0.6-0.9)	0.8 (0.6-1.0)	0.0133^d^
Oxygen requirement at rest before transplant (liter/min), median (IQR)^h^	4.0 (2-6)	4.0 (2-6)	4.0 (2-6)	0.0824^d^
Six-minute walking distance at time of listing (m), median (IQR)^i^	930.0 (463-1,240)	872.0 (400-1,204)	895.0 (400-1,210)	0.5474^d^
ECMO at time of transplant, *n* (%)^j^	6 (5.0%)	31 (5.9%)	37 (5.8%)	0.8297^b^
Ventilator at time of transplant, *n* (%)^j^	14 (11.7%)	35 (6.7%)	49 (7.6%)	0.0837^b^
Post-transplant				
Hospital length of stay (days), median (IQR)^k^	30.0 (17-57)	18.0 (13-29)	20.0 (13-32)	<0.0001^d^
Bronchiolitis obliterans syndrome, *n* (%)^l^	20 (23.5%)	152 (39.1%)	172 (36.3%)	0.0086^b^
Post-transplant dialysis prior to discharge, *n* (%)^m^	30 (25.2%)	38 (7.7%)	68 (11.1%)	<0.0001^b^
Severe renal dysfunction, *n* (%)^n^	23 (25.6%)	62 (15.1%)	85 (17.0%)	0.0205^b^
Causes for graft failure^o^				0.0278^b^
Acute rejection, *n* (%)	0 (0.0%)	6 (5.3%)	6 (4.6%)	
Chronic rejection, *n* (%)	3 (16.7%)	51 (45.1%)	54 (41.2%)	
Primary nonfunction	6 (33.3%)	14 (12.4%)	20 (15.3%)	
Other	9 (50.0%)	42 (37.2%)	51 (38.9%)	

Abbreviations: A1ATD, alpha-1 antitrypsin deficiency; BMI, body mass index; CF, cystic fibrosis; Con-AbLTx, concomitant abdominal/lung transplant; COPD, chronic obstructive pulmonary disease; IIP, idiopathic interstitial pneumonia; IPAH, idiopathic pulmonary arterial hypertension; IQR, interquartile range; LTx, lung transplant; ECMO, extracorporeal membrane oxygenation.

*p*-Values reflect 2-group comparisons between Con-AbLTx and LTx-only. Severe renal dysfunction defined as creatinine >2.5 mg/dl, dialysis, or renal transplant.

a Three hundred and seventy-eight cases removed for missing data.

b Fisher exact.

c Nine hundred cases removed for missing data.

d Wilcoxon rank sum.

e Three hundred and nineteen cases removed for missing data.

f Seven hundred and eleven cases removed for missing data.

g Three hundred and twenty-two cases removed for missing data.

h Four hundred and eighteen cases removed for missing data.

i Six hundred and seventy-three cases removed for missing data.

j Three hundred and thirty-two cases removed for missing data.

k Three hundred and ninety-five cases removed for missing data.

l Five hundred and one cases removed for missing data.

m Three hundred and sixty-five cases removed for missing data.

n Four hundred and seventy-five cases removed for missing data.

o Eight hundred and forty-four cases removed for missing data.

**Table 3:** Lung Diagnosis for Transplant Category Subgroups

Lung diagnosis category	LTx-only (*n* = 780)	Lung/kidney (*n* = 51)	Lung/kidney/liver (*n* = 4)	Lung/liver (*n* = 135)	Lung/liver/pancreas (*n* = 2)	Lung/pancreas (*n* = 3)	Total (*n* = 975)
A1ATD	36 (4.6%)	1 (2.0%)	0 (0.0%)	9 (6.7%)	0 (0.0%)	0 (0.0%)	46 (4.7%)
CF	409 (52.4%)	14 (27.5%)	1 (25.0%)	76 (56.3%)	2 (100.0%)	3 (100.0%)	505 (51.8%)
COPD	39 (5.0%)	6 (11.8%)	0 (0.0%)	3 (2.2%)	0 (0.0%)	0 (0.0%)	48 (4.9%)
CTD	5 (0.6%)	1 (2.0%)	0 (0.0%)	0 (0.0%)	0 (0.0%)	0 (0.0%)	6 (0.6%)
IIP	133 (17.1%)	11 (21.6%)	3 (75.0%)	20 (14.8%)	0 (0.0%)	0 (0.0%)	167 (17.1%)
ILD-not IIP	32 (4.1%)	4 (7.8%)	0 (0.0%)	4 (3.0%)	0 (0.0%)	0 (0.0%)	40 (4.1%)
IPAH	48 (6.2%)	3 (5.9%)	0 (0.0%)	10 (7.4%)	0 (0.0%)	0 (0.0%)	61 (6.3%)
LAM/tuberous sclerosis	9 (1.2%)	3 (5.9%)	0 (0.0%)	0 (0.0%)	0 (0.0%)	0 (0.0%)	12 (1.2%)
Non–CF-bronchiectasis	16 (2.1%)	2 (3.9%)	0 (0.0%)	1 (0.7%)	0 (0.0%)	0 (0.0%)	19 (1.9%)
OB	6 (0.8%)	1 (2.0%)	0 (0.0%)	1 (0.7%)	0 (0.0%)	0 (0.0%)	8 (0.8%)
Other	13 (1.7%)	1 (2.0%)	0 (0.0%)	3 (2.2%)	0 (0.0%)	0 (0.0%)	17 (1.7%)
PH-not IPAH	25 (3.2%)	4 (7.8%)	0 (0.0%)	5 (3.7%)	0 (0.0%)	0 (0.0%)	34 (3.5%)
Retransplant	6 (0.8%)	0 (0.0%)	0 (0.0%)	1 (0.7%)	0 (0.0%)	0 (0.0%)	7 (0.7%)
Sarcoidosis	3 (0.4%)	0 (0.0%)	0 (0.0%)	2 (1.5%)	0 (0.0%)	0 (0.0%)	5 (0.5%)

Abbreviations: A1ATD, alpha-1 antitrypsin deficiency; CF, cystic fibrosis; CTD, connective tissue disease; COPD, chronic obstructive pulmonary disease; IIP, idiopathic interstitial pneumonia; IPAH, idiopathic pulmonary arterial hypertension; ILD, interstitial lung disease; LAM, lymphangioleiomyomatosis; LTx, lung transplant; OB, obliterative bronchiolitis.

Regarding pretransplant factors, no significant differences were identified between the Con-AbLTx and LTx-only recipients in chronic steroid use (30.3% vs 37.7%, *p* = 0.1372), cigarette pack years at time of listing (5.0 vs 2.5 years, *p* = 0.1293), history of diabetes at time of listing (38.2% vs 28.3%, *p* = 0.1384), hospitalization rates within 90 days before transplant admission (43.4% vs 47.4%, *p* = 0.6460), most recent oxygen requirement at rest (4 liter/min, *p* = 0.0824), and six-minute walking distance at time of listing (930 vs 872 m, *p* = 0.5474). Con-AbLTx patients had a higher recent serum creatinine before transplant (0.9 vs 0.8 mg/dl, *p* = 0.0133). LTx-only patients were more often given immunosuppressive medication for maintenance or antirejection at the time of discharge (98.7% vs 92.8%, *p* = 0.0008). A similar number of Con-AbLTx patients were on extracorporeal membrane oxygenation at time of transplant as LTx-only patients (5.0% vs 5.9%, *p* = 0.8297). The increased need for ventilator support among Con-AbLTx at time of transplant between the 2 groups approached statistical significance at 11.7% compared to 6.7% in LTx-only patients (*p* = 0.0837).

In the postoperative period, no significant differences were identified between Con-AbLTx and LTx-only recipients in the rate of stroke before discharge (5.1% vs 3.1%, *p* = 0.2658). Con-AbLTx patients were more likely to require dialysis before discharge (25.2% vs 7.7%, *p* < 0.0001) and more often developed severe renal dysfunction (25.6% vs 15.1%, *p* = 0.0205). Con-AbLTx patients required a longer hospital length of stay post-transplant of 30 days compared to 18 days in LTx-only patients (*p* < 0.0001). Con-AbLTx recipients were less likely to develop bronchiolitis obliterans syndrome (BOS) compared to LTx-only counterparts (23.5% vs 39.1%, *p* = 0.0086). In terms of graft failure, LTx-only patients were more likely to develop acute rejection (5.3% vs 0%, *p* = 0.0278) and chronic rejection (45.1% vs 16.7%, *p* = 0.0278). The cause of death is detailed in Table 4.

**Table 4:** Cause of Death Following Transplant

Cause of death category	LTx-only (*n* = 780)	Lung/kidney (*n* = 51)	Lung/kidney/liver (*n* = 4)	Lung/liver (*n* = 135)	Lung/liver/pancreas (*n* = 2)	Lung/pancreas (*n* = 3)	Total (*n* = 975)
Missing	490	29	3	92	0	0	614
Acute rejection	4 (1.4%)	0 (0.0%)	0 (0.0%)	1 (2.3%)	0 (0.0%)	0 (0.0%)	5 (1.4%)
Cardiovascular	13 (4.5%)	0 (0.0%)	0 (0.0%)	4 (9.3%)	0 (0.0%)	0 (0.0%)	17 (4.7%)
CMV	1 (0.3%)	0 (0.0%)	0 (0.0%)	0 (0.0%)	0 (0.0%)	0 (0.0%)	1 (0.3%)
Graft failure	56 (19.3%)	3 (13.6%)	0 (0.0%)	6 (14.0%)	0 (0.0%)	1 (33.3%)	66 (18.3%)
Infection, non-CMV	60 (20.7%)	7 (31.8%)	0 (0.0%)	9 (20.9%)	0 (0.0%)	2 (66.7%)	78 (21.6%)
Lymphoma	6 (2.1%)	0 (0.0%)	0 (0.0%)	1 (2.3%)	0 (0.0%)	0 (0.0%)	7 (1.9%)
Malignancy, other	20 (6.9%)	4 (18.2%)	0 (0.0%)	2 (4.7%)	0 (0.0%)	0 (0.0%)	26 (7.2%)
Multiple organ failure	27 (9.3%)	5 (22.7%)	0 (0.0%)	7 (16.3%)	1 (50.0%)	0 (0.0%)	40 (11.1%)
OB/BOS	55 (19.0%)	0 (0.0%)	0 (0.0%)	2 (4.7%)	0 (0.0%)	0 (0.0%)	57 (15.8%)
Other	40 (13.8%)	2 (9.1%)	0 (0.0%)	10 (23.3%)	1 (50.0%)	0 (0.0%)	53 (14.7%)
Technical	8 (2.8%)	1 (4.5%)	1 (100.0%)	1 (2.3%)	0 (0.0%)	0 (0.0%)	11 (3.0%)

Abbreviations: BOS, bronchiolitis obliterans syndrome; CMV, cytomegalovirus; LTx, lung transplantation; OB, obliterative bronchiolitis.

**Figure 2:**
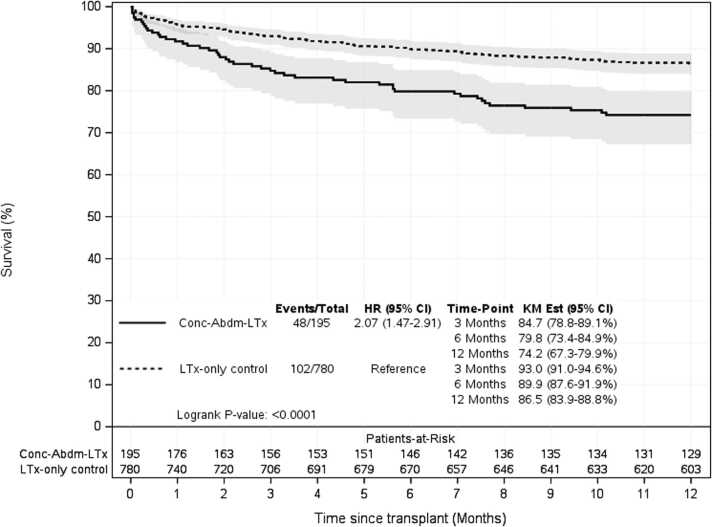
One-year survival of Con-AbLTx vs LTx-only. Con-AbLTx, concomitant abdominal organ transplant of the liver, kidney, or pancreas with lung transplant; KM, Kaplan-Meier; LTx, lung transplantation.

**Figure 3:**
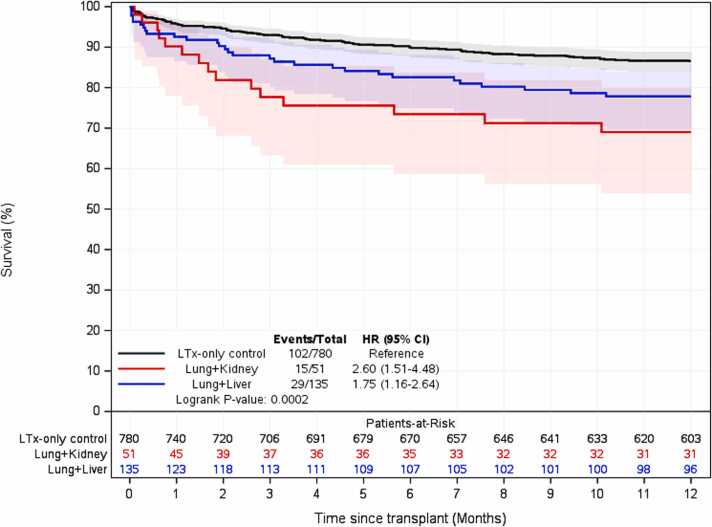
One-year survival of LTx-only, lung/liver, and lung/kidney. LTx, lung transplantation.

**Figure 4:**
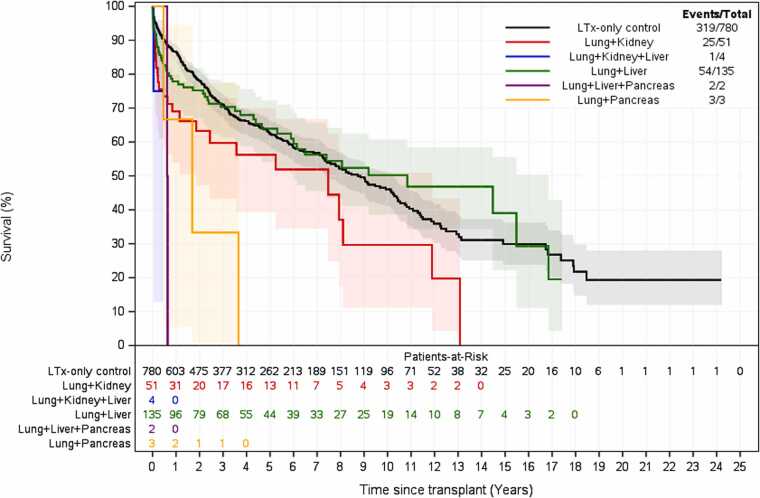
Overall survival of Con-AbLTx vs LTx-only, with breakdown by secondary organ. Con-AbLTx, concomitant abdominal organ transplant of the liver, kidney, or pancreas with lung transplant; LTx, lung transplantation.

**Figure 5:**
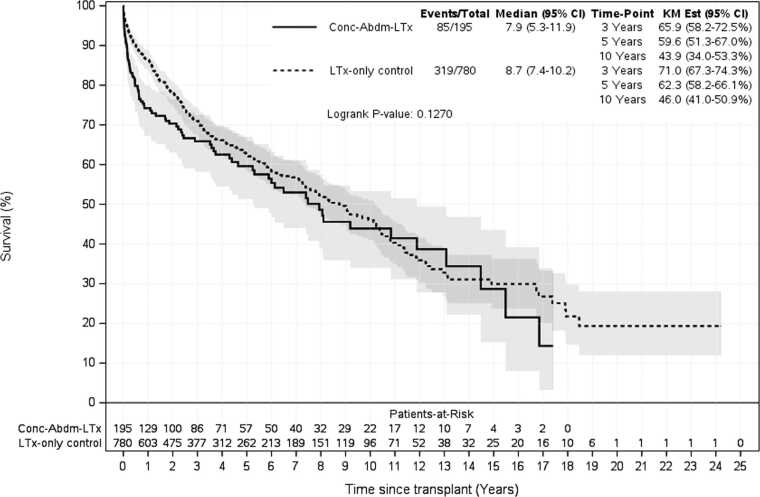
Overall survival of Con-AbLTx vs LTx-only. Con-AbLTx, concomitant abdominal organ transplant of the liver, kidney, or pancreas with lung transplant; KM, Kaplan-Meier; LTx, lung transplantation.

**Figure 6:**
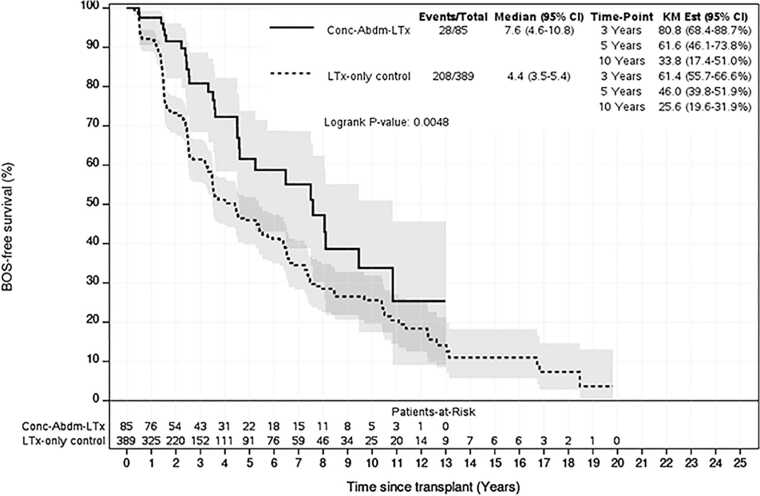
BOS-free survival of Con-AbLTx vs LTx-only. BOS, bronchiolitis obliterans syndrome; Con-AbLTx, concomitant abdominal organ transplant of the liver, kidney, or pancreas with lung transplant; KM, Kaplan-Meier; LTx, lung transplantation.

Survival analysis demonstrated that overall, Con-AbLTx recipients experienced higher mortality in the first year post-transplant compared to LTx-only patients (74% vs 87% survival, *p* < 0.001) (Figure 2). Among the breakdown of concomitant abdominal organs included in this study, concomitant lung/kidney transplant recipients demonstrated the greatest mortality relative to LTx-only within the first year (hazard ratio 2.6, *p* < 0.001) (Table 5, Figure 3). Of the concomitant transplants, lung/liver recipients had the greatest survival over time compared to other simultaneous organ pairings, although some subsets had a limited number of transplants performed for analysis (Figure 4). However, long-term survival at 10 years post-transplant showed no statistically significant difference between LTx-only recipients and Con-AbLTx at large (46% survival vs 43.9%, respectively, *p* = 0.1270) (Figure 5). Among the patients in this study (*n* = 975), 51% of patients had missing data required to construct BOS-free survival (50% missing for LTx-only controls; 56% missing for Con-AbLTx). Despite the limited data and considering that only patients with observed BOS status and time were considered for BOS-free survival, it is interesting to note that Con-AbLTx patients showed significantly favorable outcomes where BOS-free survival was improved compared to LTx-only controls (*p* = 0.0048) (Figure 6).

**Table 5:** Cox Analysis of 1-Year Survival Compared to LTx-Only Control Group

Variable	Lung/liver vs LTx-only^a^	Lung/kidney vs LTx-only^a^	Con-AbLTx vs LTx-only^b^
HR (95% CI)	1.75 (1.16-2.64)	2.60 (1.51-4.48)	2.07 (1.47-2.91)

Abbreviations: CI, confidence interval; HR, hazard ratio.

a In a single unadjusted model with 3 groups (lung/liver, lung/kidney, and LTx-only).

b In a single unadjusted model with 2 groups (Con-AbLTx [lung/liver + lung/kidney combined] and LTx-only).

## Discussion

Concomitant abdominal organ transplant with lung transplant is a relatively infrequent entity, as reflected by the sparse literature surrounding this topic. Here, we provide a contemporary and comprehensive analysis of the ISHLT Transplant database describing outcomes in simultaneous organ transplant. Comparable long-term survival of Con-AbLTx and LTx-only recipients and lower rates of rejection in Con-AbLTx highlight the utility of simultaneous transplant for select patients.

Current literature regarding simultaneous abdominal organ and lung transplants tends to solely focus on combined lung/liver transplantation. However, the benefit of simultaneous transplant remains highly debated. Studies by both Purvis and Freischlag have identified liver/lung recipients to have inferior survival compared to liver-alone recipients but similar survival to LTx-only recipients with a similar degree of liver dysfunction.^6^, ^12^ Given the heterogeneity in demonstrable benefit of combined liver/lung transplantation and the increasing organ shortage, guidance in the form of medical necessity criteria is paramount. The Organ Procurement and Transplantation Network (OPTN) has presented such guidelines for simultaneous lung/kidney transplants, the frequency of which is increasing despite a relatively low census compared to the overall number of performed transplants.^8^, ^13^ Simultaneous lung/kidney patients trend toward higher first-year mortality post-transplant.^14^ In a broader perspective on simultaneous thoracic and abdominal transplantation, Wolf et al discuss the nature of simultaneous thoracic-abdominal organ recipients to be sicker at time of transplant, often requiring hospital or intensive care unit-level admission.^9^ Despite having a higher waitlist mortality, simultaneous thoracic-abdominal organ recipients demonstrated no statistically significant difference in 5-year survival compared to thoracic-only recipients.^9^ These results from the OPTN database analysis performed by Wolf et al suggest that the underlying baseline characteristics and comorbidities of transplant recipients merit further investigation.^9^

The results of our study continue to build upon these conclusions and provide granularity on the outcomes of dual transplantation. Con-AbLTx cases demonstrated increased rates of postoperative complications such as severe renal dysfunction, need for dialysis, and longer hospital stay compared to LTx-only recipients, highlighting the morbidity and physiological stress of a dual transplantation procedure. In addition, the increased mortality in the first year post-transplant in Con-AbLTx echoes the existing literature and further emphasizes the complexity of a dual transplant.^15^ However, it is likely that patients submitted to concomitant transplant are sicker at time of transplant, as partially evidenced by a higher serum creatinine level before transplant in Con-AbLTx cases, contributing to poorer postoperative outcomes.

Concomitant liver/lung transplants constituted the largest proportion of simultaneous transplants analyzed in this study, contributing also to CF being the most common diagnosis identified. We hypothesize that the lower rates of BOS and rejection seen in Con-AbLTx cases are the results of a liver-driven protective mechanism in simultaneous transplants. This phenomenon has been described in other studies, with the liver providing immunologic protection to other simultaneously transplanted organs when the allografts are from the same donor (as in this study).^7^, ^16^ Lung/liver recipients have demonstrated a need for lower levels of tacrolimus to remain rejection free, further highlighting that the liver may provide an immunologic benefit.^7^, ^16^ Although the mechanism of this process remains unclear, it has been suggested that liver antigen-presenting cells suppress CD8+ and CD4+ T-cells.^7^, ^16^ Alternatively, the liver may neutralize lymphocytotoxic antibodies and the increased antigen load in combined transplant in itself may be beneficial.^7^, ^16^ Thus, these effects may contribute to lower rates of rejection seen across the grouping of Con-AbLTx cases in this study. This is in contrast to the reports of Freischlag et al which indicate no difference in acute rejection rates between concomitant liver/lung recipients and lung-only recipients with a similar degree of liver dysfunction before transplant, a variance which may be attributed to the use of the OPTN database as compared to ISHLT.^12^

Simultaneous lung/kidney transplants comprised the second most common concomitant transplant analyzed in our study. Overall, Con-AbLTx demonstrated higher rates of postoperative dialysis and severe renal dysfunction compared to LTx-only counterparts, likely due to the contributions of the lung/kidney subgroup. These results may be due in part to the effects of delayed graft function (DGF) in combined lung/kidney transplant. In renal transplantation alone, the incidence of DGF varies widely based on several variables, anywhere from 1.6% to 55%, largely depending on the type of organ donation.^17^ DGF is associated with a higher need for dialysis and a prolonged hospital length of stay in the postoperative period, both of which are seen in our grouping of Con-AbLTx cases.^17^ In addition, DGF is associated with increased mortality in renal transplant recipients.^17^ Thus, the particularly high mortality rate of combined lung/kidney recipients within the first year post-transplant may be due, in part, to occurrence of DGF in combined lung-kidney recipients.^17^ Interestingly, LTx recipients who receive sequential renal transplant demonstrate improved duration and quality of life compared to LTx-only recipients on dialysis or on the renal transplant waitlist.^18^, ^19^ This outcome difference based on the timing of renal transplant may suggest that certain abdominal organs, such as the kidney, should be transplanted in a sequential rather than concomitant manner within a select patient population.

Finally, our study explored rare simultaneous transplants such as lung/kidney/liver, lung/pancreas, and lung/liver/pancreas, although we are quite limited by sample size. Current literature regarding these transplant combinations is limited, largely to case reports. Lung/pancreas recipients with a diagnosis of CF demonstrated improvement in oxygen, insulin, and pancreatic enzyme requirements up to 14 months post-transplant, although their postoperative courses were complicated by difficulties in wound healing, bleeding, and thrombosis.^1^ Barbas et al reported similar improvements in both endocrine and exocrine functions of the pancreas 1 year after lung/liver/pancreas transplant for CF.^2^

While simultaneous transplantation of the lung with an abdominal organ is a potential solution to the systemic nature of specific illnesses, there are several clinical and ethical considerations for concomitant lung-abdominal organ transplant. Organ allocation in the United States primarily prioritizes patients with the greatest medical urgency while maximizing benefit to all.^9^ However, with a dual allocation listing, the risk of waitlist mortality for simultaneous transplant candidates may increase despite differences in baseline illness, and odds of transplant may be lower.^12^ Despite observing no significant difference in mortality in lung-liver recipients as compared to lung-only, dual allocation candidates are often deemed to have greater medical urgency than single allocation candidates and are permitted for liver match when a lung match is procured even when the urgency of lung failure is greater than liver failure.^12^ The complexity of concomitant lung-abdominal organ transplant considerations in the midst of limited organ availability is no doubt a challenge during transplant evaluation.

This study is not without its limitations. Inherent to database research, potential bias may arise due to variations in institutional data entry and lack of standardization may contribute. Transplant outcomes are also often multifactorial and not well-captured by database studies. For example, it is likely that differences in immunosuppressive regimens may factor into the results seen in our study; however, these medications are difficult to ascertain from database review. In addition, intraoperative data, such as transplant strategy, were not available through the database. Although propensity score analysis is typically used for such database analyses, this additionally introduces bias into the study. Our combined analysis of concomitant abdominal organ/lung recipients is an alternative to detailed subgroup analysis by secondary organ, limiting interpretation of our results. Combined analysis for outcomes other than survival was chosen given the low sample size of several subgroups.

## Conclusion

In conclusion, we demonstrate that Con-AbLTx in patients with multi–end-stage organ dysfunction results in increased rates of postoperative complications and higher first-year mortality with similar survival rates thereafter compared to LTx-only recipients. However, the lower rates of rejection in Con-AbLTx should be further investigated, though likely due to the nature of the liver as an immunologic organ. As analysis of preoperative and intraoperative variables is limited in our study, it is difficult to ascertain specific patient criteria or transplant strategies to guide patient selection for combined transplant. Strict donor selection is also crucial to successful combined transplant, as combined transplant recipients may have more difficulty tolerating organ graft dysfunction compared to LTx-only patients. Given limited organ availability and ethical considerations of simultaneous transplant, a call to arms is also needed to standardize criteria for simultaneous transplantation to maximize outcomes and ethical organ allocation.

## Author contributions

R.W.: conceptualization, data curation, formal analysis, investigation, writing—original draft, writing—review and editing; J.A.J.: formal analysis, writing—original draft, writing—review and editing; J.M.F.: writing—review and editing; B.L.: conceptualization, data curation, formal analysis, investigation, methodology, software, writing—review and editing; B.A.: supervision, writing—review and editing; A.O.: supervision, writing—review and editing; J.D.: supervision, writing—review and editing; P.R.D.S.: conceptualization, data curation, formal analysis, investigation, supervision, writing—review and editing.

## Disclosure statement

The authors declare that they have no known competing financial interests or personal relationships that could have appeared to influence the work reported in this paper.

This research did not receive any specific grant from funding agencies in the public, commercial, or not-for-profit sectors.

## Data Availability

The data have been provided by the ISHLT Thoracic Organ Transplant Registry. The interpretation and reporting of these data are the responsibility of the user(s).
